# Congenital hyperinsulinism in clinical practice: From biochemical pathophysiology to new monitoring techniques

**DOI:** 10.3389/fped.2022.901338

**Published:** 2022-09-23

**Authors:** Mariangela Martino, Jacopo Sartorelli, Vincenza Gragnaniello, Alberto Burlina

**Affiliations:** ^1^Department of Child and Mother, University of Padua, Padua, Italy; ^2^Division of Inborn Metabolic Disease, Department of Pediatrics, University Hospital Padua, Padua, Italy

**Keywords:** hypoglycemia, hyperinsulinism, glucose, CGM, inborn error of metabolism

## Abstract

Congenital hyperinsulinism comprises a group of diseases characterized by a persistent hyperinsulinemic hypoglycemia, due to mutation in the genes involved in the regulation of insulin secretion. The severity and the duration of hypoglycemic episodes, primarily in the neonatal period, can lead to neurological impairment. Detecting blood sugar is relatively simple but, unfortunately, symptoms associated with hypoglycemia may be non-specific. Research in this field has led to novel insight in diagnosis, monitoring and treatment, leading to a better neurological outcome. Given the increased availability of continuous glucose monitoring systems that allow glucose level recognition in a minimally invasive way, monitoring the glycemic trend becomes easier and there are more possibilities of a better follow-up of patients. We aim to provide an overview of new available technologies and new discoveries and their potential impact on clinical practice, convinced that only with a better awareness of the disease and available tools we can have a better impact on CHI diagnosis, prevention and clinical sequelae.

## Introduction

During the neonatal and infant period, hyperinsulinemic hypoglycemia (HH) is the most common and severe etiology of persistent hypoglycemia. Hypoglycemia is a common metabolic finding in pediatric age that in severe cases can lead to permanent neurological sequelae, with a broad spectrum of causes (1). Low blood glucose (<2 mmol/L), may result in seizures and brain damage, which lead to developmental delay, motor and learning disabilities, and exceptionally to death (2, 3). There is no strict biochemical definition of hypoglycaemia. Clinical hypoglycemia is defined as a plasma glucose concentration low enough to cause symptoms and/or signs of impaired brain function but operative thresholds should be considered. Based on international guidelines, standard of care is treating neonates aged <48 h with a threshold of 2.8 mmol/L, those aged >48 h with a threshold of 3.3 mmol/L. Older infants and children should be treated when blood glucose level is <3.9 mmol/L (4).

Two principal forms of HH are recognized, a transient and a permanent one. Transient HH is usually associated with risk factors such as intrauterine growth restriction (IUGR), perinatal asphyxia, prematurity, erythroblastosis fetalis, maternal diabetes mellitus, maternal medications (sulfonylurea, beta-blockers), intravenous glucose infusion during labor or associated with various overgrowth syndromes like Beckwith-Wiedemann or metabolic conditions such as congenital disorders of glycosylation (5–7).

Congenital hyperinsulinism (CHI) is a rare, orphan disease with an estimated incidence of 1 in ~50,000 live births. It comprises a group of diseases characterized by a persistent hyperinsulinemic hypoglycemia, due to mutation in the genes involved in the regulation of insulin secretion.

Symptoms of hypoglycemia can range from non-specific adrenergic symptoms (poor feeding, hunger, palpitations, sweating) to life-threatening, neuroglycopenic symptoms (seizures, unconsciousness, lethargy, coma and even death) (7).

Newborns with HH may be macrosomic due to intrauterine hyperinsulinemia. However, the absence of macrosomia does not exclude CHI. Hypertrophic cardiomyopathy and hepatomegaly are also observed in some patients (8).

HH is associated with suppressed production of ketones, which serve as an alternative fuel for the brain when glucose supply is low, leading to a potential damage to nervous cells and to neurodevelopmental disabilities. It is therefore essential to promptly detect it and to start a correct therapy (9).

Of note, expanded neonatal screening that is covering almost 50 different metabolic diseases with acute presentation in neonatal period does not allow to recognize this disorder.

In this review, we present recent insights about CHI, starting from diagnosis and pathophysiology to neurodevelopmental outcomes, focusing on the impact of new technologies in this field.

## Biochemical pathways and pathogenesis

Novel genetic techniques allowed a better understanding of molecular mechanisms involved in the regulation of insulin secretion and the discovery of mutations in various genes which can cause CHI.

**Figure 1:**
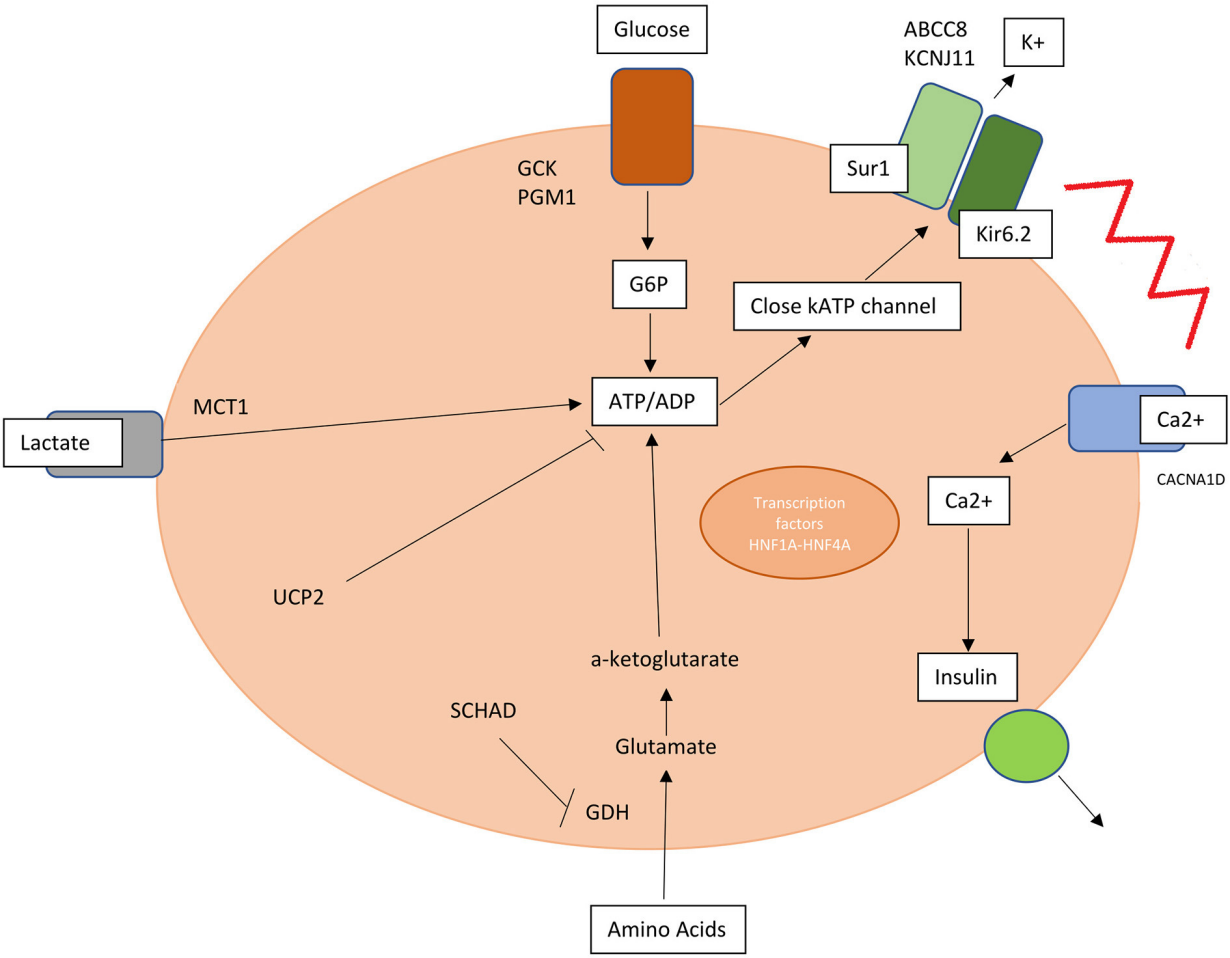
Mutations in genes involved in insulin release and production are responsible for hyperinsulinism. SUR1, sulphonylurea receptor 1; Kir6.2, inwardly rectifying potassium channel 6.2; K, potassium; MCT1, monocarboxylate transporter 1; G6P, glucose 6 phosphate; PGM1, phosphoglucomutase 1; UCP2, mitochondrial uncoupling protein 2; SCHAD, short-chain L-3-hydroxyacyl-CoA dehydrogenase; HNF1A and 4A, hepatocyte nuclear factor 1A and 4A; Ca^2+^, calcium.

A key role is hereby assumed by mutations in genes involved in the regulation of insulin secretion from pancreatic β-cells (Table 1, Figure 1).

**Table 1:** Principal genes involved in congenital hyperinsulinism.

**Function**	**Genes**	**Inheritance**	**Diazoxide responsiveness**	**Clinical features**
KATP channel proteins	ABCC8-KCNJ11	Autosomal recessive	No	Diffuse hyperinsulinism
	ABCC8-KCNJ11	Autosomal dominant	Yes/No	Diffuse hyperinsulinism
	ABCC8-KCNJ11	Monoallelic paternal	Yes/No	Focal hyperinsulinism
Ion and solute transporters	KCNQ1	Autosomal dominant/recessive	Yes	Hereditary long QT syndrome, deafness, gastrointestinal defects
	CACNA1D	Autosomal dominant	Yes	Heart defects and severe hypotonia
	SLC16A1	Autosomal dominant	Yes	Exercise-induced hyperinsulinism
Trancription factors	HNF1A	Autosomal dominant	Yes	MODY 1
	HNF4A	Autosomal dominant	Yes	MODY 3
	FOXA2	Autosomal dominant	Yes	Hypopituitarism
	EIF2S3	X-linked recessive	Yes	Hypopituitarism, transient postprandial hypoglycemia
Enzymes and regulators of vescicle release	GLUD1	Autosomal dominant	Yes	Hyperinsulinism hyperammonemia syndrome
	GCK	Autosomal dominant	Yes/no	
	HADH	Autosomal recessive	Yes	
	UCP2	Autosomal dominant	Yes	
	HK1	Autosomal dominant	Yes	
	PMM2	Autosomal recessive	Yes	Polycystic kidney disease
	PGM1	Autosomal recessive	No	Hypertransaminasemia, hyperCKemia, cleft palate, growth delay

KATP channel is an ATP-sensitive potassium channel situated on the membrane of pancreatic beta-cell formed by two types of subunits: four inward-rectifying potassium channel pore-forming (Kir6.2) subunits and four high-affinity sulfonylurea receptor 1 (SUR 1) subunits. This channel transduces glucose metabolism of the cell into membrane depolarization. Glucose entry is mediated by GLUT2 transporter and once inside the membrane this substrate is transformed into glucose-6-phosphate by glucokinase (GCK) and transformed through glycolysis into ATP.

An increased ATP/ADP ratio leads to KATP closure, cell depolarization and calcium influx into the cell *via* voltage gated calcium channels triggering insulin release. Conversely a decrease in blood glucose level leads to KATP opening and membrane hyperpolarization (10, 11).

CHI can be caused by defects in genes encoding pancreatic KATP channels (ABCC8 and KCNJ11) and other channels/transporter proteins (KCNQ1, CACNA1D, and SLC16A1), in enzymatic and vesicle-trafficking regulator genes (GLUD1, GCK, HADH, UCP2, HK1, PMM2, and PGM1) and transcription factors genes (HNF1A, HNF4A, FOXA2, and EIF2S3) (12).

Inactivating KATP channel mutations are responsible for the most common and severe type of HI, causing both diffuse and focal forms. The diffuse form affects all the pancreatic islets and constitutes 60–70% of all the KATP-CHI cases. It is mainly inherited in an autosomal recessive manner, while autosomal dominant variants cause milder forms. This is due to inactivating mutations in ABCC8 or KCNJ11 and is usually medically unresponsive and may require a near-total pancreatectomy. The focal form constitutes 30–40% of all CHI cases, it is mainly caused by a monoallelic mutation in the same gene with paternal inheritance and a somatic loss of the maternal 11p15 in a small region of the pancreas. The atypical forms consist of a mosaic pattern of the above two forms (diffuse or focal) and have shrunken or enlarged islets (13–15).

## Diagnostic workup

The clinical spectrum of HH is wide and patients may also present with normal birth weight, require minimal dextrose support, or present outside of infancy.

The classic presentation of CHI is at birth. Infants with HH require a high glucose infusion rate, >8 mg/kg/min (normally 4–6 mg/kg/min). The threshold for obtaining diagnostic data (often referred to as the “critical sample”) to measure major metabolic fuels and counter regulatory hormones and to confirm a diagnosis of hypoglycemia is <50 mg/dl (2.8 mmol/L). This has to be done before initiating treatment, either during the initial acute episode or during a supervised diagnostic fast (4).

The critical blood sample should be tested for plasma glucose, blood gas, lactate, ammonia, beta-hydroxybutyrate (BOHB), free fatty acids (FFAs), plasma amino acids, acyl-carnitine profile, free and total carnitine, insulin, C-peptide, cortisol, growth hormone (Supplementary Figure 1).

The critical urine sample should be obtained at the time of the acute event and tested for organic acids and ketones.

Elevated serum insulin at the time of hypoglycemia is the most direct method of detection of hyperinsulinism, but, even if uncommon with high sensitivity assays, an undetectably low insulin concentration (because of the hemolysis and release of insulin-degrading enzyme) does not exclude the diagnosis (14).

Unlike insulin, C-peptide is not subject to hepatic clearance or hemolysis and may more accurately reflect beta-cell function. C-peptide threshold of ≥0.5 ng/ml provided the best combination of sensitivities and specificities for this biomarker.

Because insulin inhibits hepatic ketogenesis, inappropriate suppression of ketones and FFA has been proposed as a marker for excess insulin (BOHB <1.8 mM and FFA <1.7 mM) (10, 14).

An inappropriate rise in plasma glucose (Δ ≥ 1.5 mmol/L) following an injection of glucagon provides additional evidence of insulin excess because insulin inhibits hepatic glycogenolysis (16). Suppressed insulin-like growth factor binding protein-1 (IGFBP-1), <110 ng/ml, has been proposed as a supplementary marker of excess insulin activity (17). Another recent study showed that in children with HH there is a decreased C10:1 and increased threonine level, reflecting the insulin ketogenesis inhibition effect (18).

In HH there are also suppressed branch chain (leucine, isoleucine and valine) amino acids, normal lactic acid and appropriate counter regulatory hormone response: cortisol >20 mcg/dl (500 nmol/L) and growth hormone >7 ng/ml.

However, recurrent hypoglycaemia even of short duration blunts the autonomic, neuroglycopaenic and glucose counter-regulatory hormonal resulting in clinically silent hypoglycaemia. For this reason in infant with CHI serum counter-regulatory hormonal responses at the time of hypoglycaemia can be all blunted (19).

An elevated serum ammonia concentration in a patient with HH is suggestive of the hyperinsulinism and hyperammonemia (HI/HA) syndrome (20). Once laboratory test results are consistent with CHI, genetic analysis should be performed in order to define the diagnosis and to guide the clinicians to a tailored therapeutic approach (13).

## Therapeutic approaches

### Acute stage

The usual initial approach to neonatal hypoglycemia (awaiting the critical sample) is to feed the baby, using either formula or breast milk. If glucose concentration is <1.4 mmol/l (25 mg/dl) in the first 4 h of life and the baby is unable to take an oral feed, intravenous dextrose (bolus 200 mg/kg followed by an infusion of around 4–8 mg/kg per min) is usually required (21). Repeated boluses should be avoided, as the bolus of glucose is a potent trigger for insulin secretion. Normoglycemia should be achieved by delivering a continuous intravenous glucose infusion starting with 6–8 mg/kg/min. Patients with HH may require >25 mg/kg/min of intravenous glucose infusion to maintain normoglycemia. In patients with elevated glucose infusion rate and fluid overload, glucagon continuous IV infusion can be considered as a therapeutic option.

Oral dextrose gel 200 mg/kg (0.5 ml/kg of 40% dextrose), in combination with feeding, is increasingly recommended as a first-line treatment for asymptomatic neonatal hypoglycemia (22).

Severe or prolonged hypoglycemia, indicated by persistently high or ongoing (≥3 days) intravenous glucose requirements, suggest underlying genetic-endocrine or metabolic pathology and further investigation is required (23).

### Chronic management

Diazoxide is useful for managing HH in many patients with CHI and it is the first line treatment in this disease. It is usually effective in all forms of CHI where the KATP channel function is intact.

The initial dose is 5 mg/kg/day, in three divided doses which can be increased up to a maximum dose of 15–20 mg/kg/day (24).

The most severe side effects that limit its use and require treatment withdrawal are fluid retention with its associated electrolyte imbalance, cardiac failure and pulmonary hypertension.

For non-responder children, it should be discontinued and the hypoglycemia treated with intravenous dextrose, while proceeding with genetic test. An infusion of glucagon may be used if needed if the glucose infusion rate is high and fluid overload is a concern (25).

The second line treatment for non-responder patients is Octreotide, a short-acting somatostatin analog (26).

The starting dose is 5 μg/kg/day given by subcutaneous injections at 6–8 h intervals with a maximum dose of 30–35 μg/kg/day. Tachyphylaxis has been reported with octreotide usage in the first 48 h requiring dose adjustments. The most common side effects, even if rare, are growth restriction, risk of necrotizing enterocolitis and gallstones (26, 27).

Based on evidence from histopathologic studies showing that the mTOR pathway is constitutively activated in pancreas of children with congenital and unresponsive HI can be treated with sirolimus preventing the need for pancreatectomy. However there are many concerns about the use of this therapy, because of potential long-term consequences from prolonged exposure, including the risk of malignancy (28–30).

Alternative to medical therapy, on selected patients, there is the opportunity of surgical intervention for the removal of islets with dysregulated insulin secretion. One of the most significant advances in the last decade was the development of ^18^F-fluoro-L-dihydroxyphenylalanine (^18^F-DOPA) positron emission tomography/computed tomography (PET/CT) scanning, which can be used to localize focal lesions, in order to guide surgeons lesions allowing the possibility of curing children with focal disease (31).

## New therapeutic approaches

Following a better knowledge of the pathophysiology and the molecular mechanisms of disease, the field is now moving toward the development of new therapeutic agents, in particular for the diazoxide resistant forms. Data from preclinical studies demonstrated that the GLP-1 receptor is constitutively activated in pancreatic islets lacking KATP channels, and that exendin-(9-39), a GLP-1 receptor antagonist, inhibits insulin secretion and increases fasting glucose (32–34).

In infants with diazoxide-unresponsive HI longer-acting somatostatin analogs (as lanreotide) and stable glucagon formulations for continuous subcutaneous administration are being evaluated in proof-of-concept clinical trials (35–38).

Ketogenic Diet is used in the short and long term treatment of drug-unresponsive GCK-HI. The neuroprotective effects of KD determined the recovery from epilepsy and intellectual disabilities and averted the need of a near-total pancreatectomy (39).

Another possible therapeutic option emerging from preclinical studies is an antibody against the insulin receptor (40).

## New technologies in glucose monitoring

Adequate monitoring of newborn at risk and very preterm infants admitted to intensive care is critical to prevent severe hypoglycemia. Current recommendations advise obtaining hourly blood glucose levels for many days, usually weeks, until optimal treatment can be safely established to enable a baby to be discharged home without the risk of hypoglycemia but blood sampling is distressing for the babies and demanding of resources (41). Continuous glucose monitoring (CGM) is increasingly being used in children with type 1 diabetes mellitus, but its use in neonatology remains limited in neonatology remains limited. Continuous interstitial glucose monitors consist of a sensor using a glucose oxidase that is inserted into the subcutaneous tissue (in the newborn/infant in the lateral part of the thigh without reports of local complications), and a recording device, which converts the electrical current generated in the sensor to a glucose concentration using an inbuilt algorithm. Needle insertion applicators are supplied with sensors, but in newborn it is preferred a manual modality to insert it.

Despite significant advances in technology (smaller size, increasingly accurate algorithms), current devices are not designed or licensed for use in babies, and there are concerns about their accuracy in assessing hypoglycemia because, like point-of-care glucometers, they are designed for use in diabetes (42–44).

Previous studies in children with HI have shown that CGM tends to under read compared with blood glucose (BG) measurements (45, 46). CGM point accuracy is usually expressed as mean absolute relative difference (MARD), defined as the mean of the absolute differences between CGM and simultaneous reference values as a percentage of the reference value. Errors of 13% or less are generally considered acceptable. Errors may be even larger at extreme glucose concentrations or when glucose concentrations are changing rapidly.

There is a lag period between changes in blood glucose concentrations and changes in the interstitial fluids, so the rapid changes in glucose concentrations that are common in newborn babies are poorly reported by continuous monitors (47).

These data mean CGM is best placed to act as an adjunct on glucose trends and to provide reassurance during periods of normoglycemia. This could potentially limit the need for such frequent blood sampling, alerting the clinician to the need for BG measurement as part of routine care (38). However, data collected using blinded CGM in infants at risk of transient neonatal hypoglycemia have demonstrated an association between clinically silent hypoglycemia, detected using CGM, and impaired executive and visuomotor outcomes at 4.5 years (47, 48).

Thus, CGM may be a valuable tool not just to support avoidance of hypoglycemia, but also to help to determine glycemic trend and the characteristics of hypoglycemic exposure, beyond simple thresholds (including length of exposure and effect of fluctuations) that affect longer term outcomes. This makes CGM data analysis a potential measured outcome in evaluating drug efficacy in this group of patients in future clinical trials (44).

Neonatal nurses typically have less CGM technology experience, and staff training would be critical to ensure that CGM was used appropriately as an adjunct to management alongside BG monitoring.

It is accepted that real-time CGM can reduce exposure to prolonged or severe hyperglycemia and hypoglycemia in diabetic patients and neonates. Although the use in neonates appears to be well tolerated, feasible and associated with better glycemic status, further studies using CGM are required to determine optimal glucose targets, strategies to obtain them, and the potential effect on long-term health outcomes (47, 49, 50).

Majority of the parents found this technology easy to use at home and convenient to monitor glycemic trends, especially during the night (51). Families noticed trends in glucose levels which motivated behavioral changes to reduce hypoglycemia with advantages outweighing disadvantages (52).

Another area in which new monitoring techniques are making room is in the control of diabetes due to pancreatic insufficiency wich follows pancreatectomy in medically unresponsive diffuse congenital HI. The bihormonal bionic pancreas (BHBP) by autonomously administering insulin and glucagon based on glucose levels detected *via* CGM systems demonstrated a trend toward an overall improvement of mean glucose and frequency of hypoglycemia in the BHBP period, consistent with previous studies in individuals with type 1 diabetes (53).

## Monitoring of complications: Neurodevelopmental outcomes

Congenital hyperinsulinism can cause recurrent and severe hypoglycemia that can lead to psychomotor retardation, learning disability and seizures. Up to 50% of children with CHI suffer from long-term neurodevelopmental disabilities and this is more evident in the diazoxide unresponsive forms (54).

The strongest associations between risk factors and severe brain injury were found for hypoglycemic seizures, lowest recorded BG <20 mg/dl and history of untreated hypoglycemia (55).

These sequelae implicate difficulties in attention (inhibition naming), verbal working memory (digit span), visual learning and memory (memory for designs), and visuomotor and sensorimotor functions (visuomotor precision and design copying) (56). This suggests a role for time spent in hypoglycemia in harming cerebral areas involved in cognitive functions, like hippocampus for memory, basal ganglia, parietal cortex and posterior white matter for visual and sensorimotor abilities (57).

The neurological outcome has a huge impact on the patient and families and a periodic assessment of neurodevelopment must be carried out since discharge (55).

Different metabolic backgrounds imply a different availability of nutrients for the brain in glucose deficit. Hyperinsulinism leads to a lack of ketone body and lactate production that are an alternative fuel for the brain and it can lead to a more consistent damage, rather than pathologies that allow production of these substrates, like glycogen storage disease. Moreover, comorbidities can lead to a more severe injury in a susceptible brain. In particular status epilepticus, hypoxic-ischemic injury, respiratory failure and infection can enhance energetic demands in a starving brain and worsen the neuroinflammatory response (58).

MRI has a better sensitivity in detecting hypoglycemic lesions than brain ultrasound, due to its definition of cortical anomalies (57). There is also evidence of a good correlation between white matter injury and more severe neurologic outcome, with a direct proportionality between extension and diffusion of lesions and cognitive impairment and seizures (59). Moreover MRI studies suggest a possible role of DWI imaging within 6 days from the hypoglycemic insult in neonates in identifying subjects with occipital diffusion restriction, a transient abnormality which can relate with a potential risk of later cortical visual deficits (60).

Functional brain imaging can also have a possible role in detecting hypoglycemic insults. Glucose uptake has been evaluated through FDG-PET, while glucose metabolism has been studied through ^13^C Magnetic Resonance Spectroscopy. Variations in regional brain blood flow or oxygenation have been evaluated through fMRI with BOLD contrast or MRI with Arterial Spin Labeling sequences. However, these functional techniques have been applied in few studies in patients with hypoglycemic episodes during therapy for type-1-diabetes, so they need further validation to be considered as a new potential tool for clinical practice (61–63).

## Conclusion and future perspectives

New technologies are impacting every field of medicine and clinicians have new tools for making better diagnosis and to treat their patients better. This is also true in hyperinsulinemic hypoglycemia diagnosis and management. In recent years, scientists are studying new biomarkers that could be more specific and sensitive. Moreover, genetics has helped in defining the molecular basis of hypoglycemia, giving insight into new pathophysiology mechanisms, like alteration in cellular vesicular transport.

Once the diagnosis has been defined, clinicians now have a broader armamentarium to try to stabilize blood glucose levels, rather than continuously feeding the child, like low release glucose solutions, precision surgery or developing pharmacological therapies that target specific molecular pathways. New technologies revolutionized disease monitoring, with the possibility of continuous glucose monitoring in a minimally invasive way.

MRI imaging could also allow the detection of hypoglycemic insult before clinical evidence, but with the limitations of capturing transient abnormalities. However, the potential impact of all these new discoveries has still to be demonstrated.

New clinical studies are needed to bring new drugs from case reports and preclinical studies into clinics and to validate new technologies in glucose monitoring in larger cohorts of children.

There is also a need to define common and better long term outcomes for clinical studies involving patients with hypoglycemia: defining a specific neurologic outcome encompassing psychomotor development requires a very long follow-up period, which is difficult to realize. Correlation between neuroimaging data and neurologic sequelae has still to be defined. Also, CGM data analysis is a promising tool for monitoring glycemic trends, but it needs further validation. All these technologies could lead to a better monitoring of therapy efficacy and to identify early subjects at risk of developing neurological disabilities, in order to achieve the best possible outcome for patients and their families.

## Author contributions

MM and JS reviewed the literature and wrote the manuscript. VG and AB supervised and reviewed the manuscript. All authors have read and agreed to the published version of the manuscript.

## Conflict of interest

The authors declare that the research was conducted in the absence of any commercial or financial relationships that could be construed as a potential conflict of interest.

## Publisher's note

All claims expressed in this article are solely those of the authors and do not necessarily represent those of their affiliated organizations, or those of the publisher, the editors and the reviewers. Any product that may be evaluated in this article, or claim that may be made by its manufacturer, is not guaranteed or endorsed by the publisher.

## Supplementary material

Supplementary Figure 1 Critical blood sample and the principal differential diagnosis.
