# Supplementary figures and images for: Congenital hyperinsulinism in clinical practice: From biochemical pathophysiology to new monitoring techniques
Source: Front Pediatr. 2022 Sep 23;10:901338. doi: 10.3389/fped.2022.901338 (PMC9538154; doi:10.3389/fped.2022.901338)

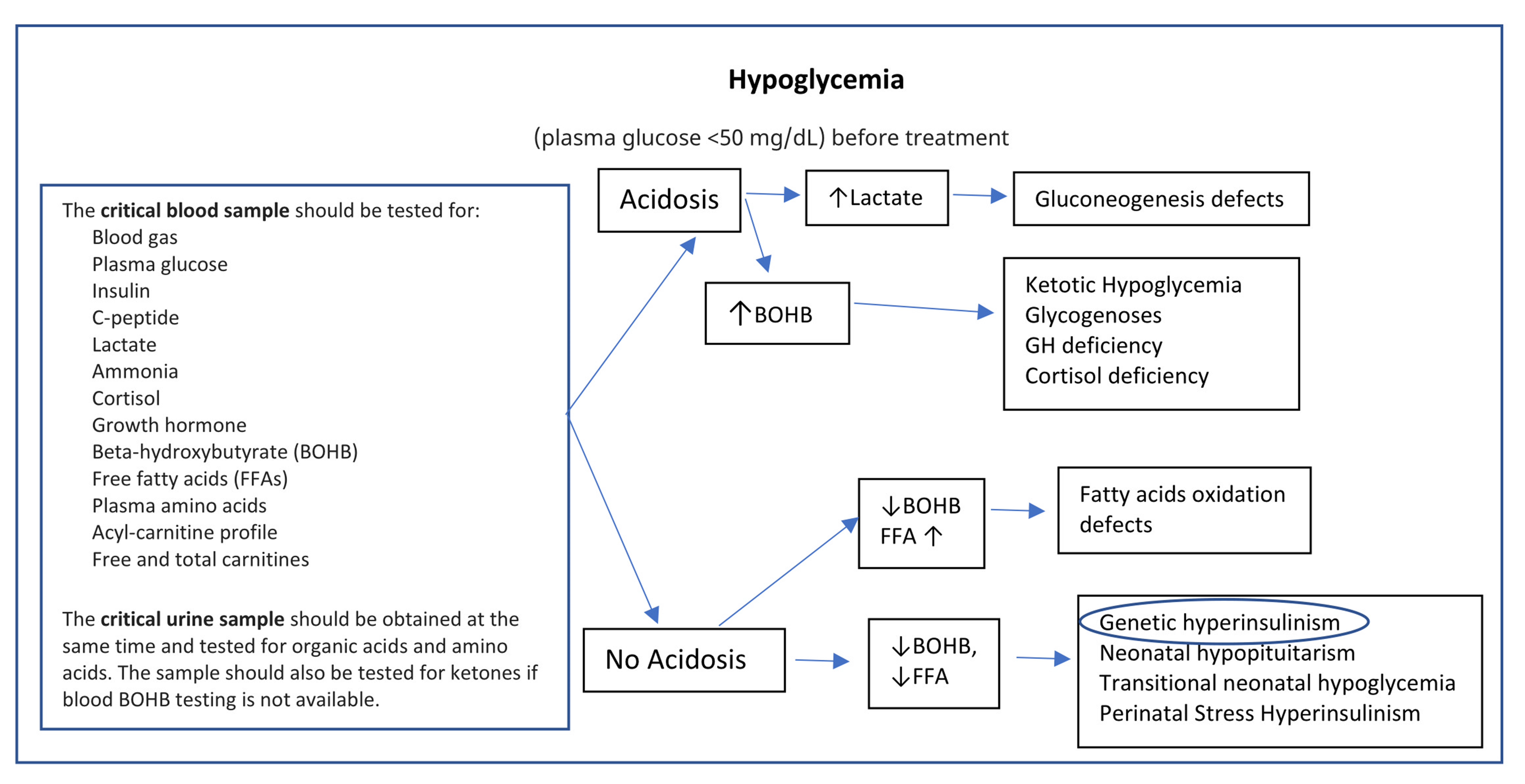

Supplement: Supplementary Figure 1 — Critical blood sample and the principal differential diagnosis. [file Image_1.TIF]
